# Allele-linked divergence in SlpA and TcdB drives distinct immune and cytotoxic responses that distinguish ST01 from non-ST01 strains in Clade 2 Clostridioides difficile

**DOI:** 10.1099/acmi.0.000994.v3

**Published:** 2025-09-10

**Authors:** Adriana Badilla-Lobo, Carlos Quesada-Gómez, Esteban Chaves-Olarte, César Rodríguez

**Affiliations:** 1 Facultad de Microbiología, Universidad de Costa Rica, San José, Costa Rica; 2 Programa de Posgrado en Microbiología, Parasitología, Química Clínica e Inmunología, Universidad de Costa Rica, San José, Costa Rica; 3 Centro de Investigación en Enfermedades Tropicales (CIET), Universidad de Costa Rica, San José, Costa Rica

**Keywords:** *Clostridioides difficile*, MLST Clade 2, phenotypic assays, virulence

## Abstract

Among the five MLST clades that define the global population structure of the bacterial pathogen *Clostridioides difficile*, Clade 2 has received special attention due to the global spread, clinical severity and hospital prevalence of ST01 strains. To identify features potentially contributing to the historically attributed higher virulence and epidemic potential of ST01 strains, we compared a range of phenotypic traits across the infection cycle between clinical Clade 2 ST01 and non-ST01 strains from ST41, ST47, ST67, ST154 and ST638. We found no significant differences in canonical virulence-associated characteristics such as spore adherence, motility, biofilm formation and resistance to a disinfectant. However, ST01 strains exhibited distinct profiles in surface layer protein A (SlpA)-mediated immune activation and toxin B (TcdB)-induced cytotoxicity that were consistent with allelic divergence. These findings highlight the need to reconsider current paradigms of Clade 2 hypervirulence and underscore the importance of allele-specific phenotypic variation in developing targeted public health strategies.

## Data Summary

No tools, software or code have been generated or are required to reproduce this work. The draft genome sequences analysed in this study are publicly available at GenBank and can be accessed through the BioProjects PRJEB3054 (SAMEA2266777 and SAMEA2266811), PRJNA224116 (SAMN04011636, SAMN04011647 and SAMN04011650) and PRJNA293889 (SAMN04011635).

## Introduction

*Clostridioides difficile*, a leading cause of diarrhoea and colitis, represents a challenge to global public health in healthcare and community settings, with its widespread prevalence correlating with substantial morbidity, mortality and socioeconomic burden [1,2].

The rising incidence of *C. difficile* infections (CDIs) and the dynamic nature of its epidemiology emphasize the urgent need to advance our understanding of *C. difficile* pathogenesis and the factors influencing disease presentation [3].

MLST studies have revealed that the global *C. difficile* population associated with humans is distributed in five clades [4,5], whereby Clade 2 strains, particularly from ST01, have garnered attention because of their epidemic potential, high toxin production, enhanced spore formation and fluoroquinolone resistance [6,9].

While data from biomodels [10] and human populations [11,12] suggest an enhanced virulence in ST01 isolates compared with their non-ST01 counterparts within *C. difficile* MLST Clade 2 [12], the evidence is still controversial [13], and the exact mechanisms substantiating this notion are yet to be elucidated.

To address this knowledge gap, we experimentally compared three ST01 isolates (two clinical isolates and the reference strain R20291) with non-ST01 clinical isolates from ST41, ST47, ST67, ST154 and ST638 in terms of phenotypic assays representing key processes across the CDI cycle, including spore adherence to intestinal cells, motility profiles, biofilm formation capacity, SlpA-mediated proinflammatory response, TcdB cytotoxicity dynamics and spore susceptibility to a highly effective sanitizing agent utilized in hospital environments.

Determining the mechanisms underlying the heightened virulence of ST01 strains can pave the way for targeted interventions and therapeutic strategies, improve diagnostics and ultimately alleviate the impact of these strains on patient outcomes and public health.

## Methods

### Strains, bacterial cultivation, identification and typing

This study used seven clinical isolates representing six sequence types (STs) from the *C. difficile* MLST Clade 2: LIBA-5700 and LIBA-5758 (ST01), LIBA-2811 (ST41), LIBA-7857 (ST47), LIBA-5757 (ST67), LIBA-6656 (ST154), and LIBA-5809 (ST638). These bacteria were obtained between 2009 and 2018 at the Anaerobic Bacteriology Research Laboratory (LIBA) from the University of Costa Rica by cultivating ethanol-shocked stool samples from CDI patients on cefoxitin-cycloserine-fructose agar plates (Oxoid) [11]. Several of these STs are globally rare, and their inclusion reflects a decade-long effort to capture the phenotypic breadth of Clade 2. Despite the modest sample size, the current collection captures unprecedented Clade 2 diversity and enables direct phenotypic comparisons that have not previously been possible.

The presumptive identification of the isolates as *C. difficile* was confirmed using the RapID 32A system (bioMérieux) [11] and an end-point PCR targeting *tpi*, *tcdA*, *tcdB*, *tcdC* and *cdtB* gene fragments [14]. For strain typing, we used PFGE [15,16] and applied FastMLST [17] to sequencing data. ORFs corresponding to the major toxins TcdA, TcdB and the binary toxin components CdtAB, together with the surface-layer protein SlpA, were retrieved from draft genomes assembled from Illumina short-read data for each strain. Allelic designations or subtypes were assigned by querying the PubMLST *C. difficile* database and DiffBase. Predicted aa sequences were aligned with MAFFT v7.490, and pairwise percentage identities were calculated with Geneious Prime® v2025.2.1 to quantify sequence divergence.

Phenotypic assays were conducted using freshly plated bacteria recovered from cryopreservation vials stored at −80 °C. Unless otherwise specified, incubations were performed at 37 °C in an anaerobic chamber (Bactron II, Shell Lab) under an atmosphere of 90% N_2_, 5 % H_2_ and 5% CO_2_. We tested two clinical ST01 isolates due to the reported variability of their phenotypes [18] and the well-known strain R20291 because of its extensively documented phenotypes. Additionally, we included the strain CD630 from MLST Clade 1 in disinfection assays for comparative purposes (see below).

### Spore suspensions

Spore suspensions were prepared according to the previously established protocols [19,20]. Briefly, the strains were grown in brain heart infusion (BHI) broth supplemented with 0.1% haemin, 0.1% vitamin K and 0.5% yeast extract for 24 h, and a dilution of these initiation cultures was subsequently spread onto trypticase soy agar plates supplemented with 0.5% yeast extract. After 7 days of incubation, the biomass on the plates was collected by flooding them with ice-cold sterile distilled water and scraping them with disposable loops. To separate spores from vegetative cells, biomass suspensions were mixed with an equal volume of Histodenz^®^ (Sigma-Aldrich) and centrifuged at 16,873 ***g*** for 10 min at room temperature. The resulting spore pellets were washed three times with PBS (0.01 M, pH 7.4) and resuspended in PBS containing 1% w/v BSA to prevent aggregation. The washed spore suspensions were stored at 4 °C for a minimum of 15 days to allow spore maturation. Following this period, the suspensions were resuspended and heat treated at 55 °C for 20 min to eliminate any remaining vegetative cells. Viable and total spore counts were determined by cultivating duplicate samples and microscopic counting of Schaeffer and Fulton-stained smears, respectively.

### Spore adherence

Spore adherence to intestinal epithelial cells was evaluated in triplicate using established protocols [19]. To this end, Caco-2 cells were seeded onto glass slides and inoculated with spores at an m.o.i. of 4 or 10, followed by incubation at 37 °C under aerobic conditions for 1 h. One group of cells was washed three times with PBS to remove unattached spores, whereas the other group was left unwashed to determine the total number of initially added spores. Cells were then lysed using 0.06% Triton X-100 for 30 min at 37 °C, and a portion of the lysates was plated onto BHI agar plates supplemented with 2% glucose, 0.5% yeast extract and 0.01% sodium taurocholate (TA). After 48 h of incubation, spore adherence was calculated using the following formula: [(final c.f.u. ml^−1^) / (initial c.f.u. ml^−1^)]×100.

### Motility assays

Motility was initially evaluated by inoculating semi-solid agar tubes prepared with BHI broth and 0.175% agar using a straight platinum needle [21]. To distinguish between swimming and swarming motility, we used cultures grown in BHI broth for 8 h and pre-reduced plates containing 25 ml of BHI broth solidified with 0.3% or 0.4% agar, respectively [22]. Swimming plates were inoculated by stab inoculation, while swarming plates were spot-inoculated. Growth diameters were recorded after 48 h of incubation [22]. Six replicates were obtained for each strain in triplicate. Strain M120 (ST11, Clade 5, non-motile) was used as a negative control [23].

### SlpA-induced proinflammatory responses

Preparations of surface layer protein (SlpA) were obtained by resuspending bacteria cultured for 18 h in in Tryptose, Yeast extract, and Thioglycollate (TYT) broth (3% Bacto tryptose, 2% yeast extract, 0.1% thioglycollate, pH 6.8) in 0.2 M glycine at pH 2 [24,25]. The yield and purity of the extracted SlpA proteins were assessed by SDS-PAGE and confirmed by Western blotting (WB) using an anti-LIBA-5758 SlpA (ST01) antisera generated in mice [25]. For signal detection, PVDF membranes were incubated with peroxidase-conjugated anti-mouse antibodies (G21040, Invitrogen), and immunological complexes were visualized using a WB substrate (Lumi-Light Plus, Roche) and a Chemidoc XRS documentation system (Bio-Rad). The identity of the protein bands obtained for LIBA-5758 was verified by MS. To evaluate the immune responses elicited by our SlpA preparations, confluent monolayers of Raw 264.7 macrophages (ATCC TIB-71) were exposed to 20 µg of SlpA protein preparations under an aerobic atmosphere containing 5% CO_2_. After 12 h of exposure, the concentration of TNF-*α* released by macrophages into the supernatants was measured using a commercial mouse-TNF-*α* ELISA kit (eBioscience). *Escherichia coli* LPS (5 µg) and a mixture of 0.2 M glycine at pH 2.2 with 2 M Tris at pH 8.0 were tested as positive and negative controls, respectively (data not shown).

### Biofilm formation

Biofilm formation was quantified using crystal violet (CV) staining of biomass grown in plastic microtiter plates [26]. Briefly, overnight cultures were prepared by incubating the strains in BHI broth supplemented with 0.1% cysteine and 0.5% yeast extract (BHI-CY). On the following day, starter cultures were diluted 1 : 100 with BHI-CY containing 0.1 M glucose [26] and passed in 1 ml aliquots in triplicate to cell culture 24-well polystyrene plates. These plates were incubated for 24, 72 and 120 h. After the specified incubation times, the wells were washed with PBS to remove unattached cells, and the biomass adhered to the plastic surfaces was stained with 1 ml of 0.2% CV per well for 30 min. Subsequently, the plates were gently washed with PBS, and CV was extracted for 30 min by adding 1 ml of methanol per well. The OD of these methanolic extracts was measured at 570 nm using a spectrophotometer (Genesys 20, Thermo Scientific). Non-inoculated wells served as negative controls. These data were normalized between 0 and 100% using the smallest and largest values, respectively.

### TcdB titration and characterization of cytopathic effects

To evaluate functional toxin production while accounting for strain-specific differences in growth kinetics, we collected culture supernatants at equivalent growth phases. Specifically, all isolates were grown anaerobically in TYT broth until reaching the early stationary phase. This standardization ensured biological comparability and minimized artefacts caused by variable growth rates. Due to the known variability of TcdB epitopes across different subtypes, we did not rely on WB for toxin quantification, as the antibodies currently available show inconsistent cross-reactivity. Instead, we used cytotoxicity titration as a functional readout, providing a more accurate measure of each isolate’s *in vivo* pathogenic potential. To do this, filter-sterilized supernatants were concentrated using StrataClean^®^ resin [27] and separated by SDS-PAGE on parallel 7.5% gels. One gel was used for immunodetection with a monoclonal anti-TcdB antibody (2CV, tgcBiomics), while the other was stained with Coomassie Brilliant Blue and used for densitometric quantification of total secreted TcdB using ImageJ software. Cytotoxic effects were tested by exposing confluent HeLa cell monolayers to serial dilutions of cell-free supernatants containing 60 µg of total protein. To confirm specificity, cytopathic effects (CPEs) were also evaluated in the presence of TcdA/TcdB-neutralizing antibodies (T5015, TechLab) [11]. The percentage of morphologically altered cells was monitored hourly over 24 h using phase-contrast microscopy and an automated cell imaging system (Cytation 3, BioTek). Cytotoxic titres were reported as the reciprocal of the dilution that produced 50% cell rounding (CPE_50_) at 24 h post-exposure.

### TcdB-induced glycosylation profiles

Monomeric GTPase glycosylation patterns induced by TcdB were investigated using WB. Briefly, HeLa cell monolayers grown in 24-well polystyrene plates were inoculated with cell-free supernatants derived from 24-h cultures in TYT broth (~250 µg of total protein). After 24 h of incubation, cells were washed with PBS and lysed with 2% SDS. Lysate proteins were separated by SDS-PAGE on 10% polyacrylamide gels, electrotransferred to PVDF membranes and probed with monoclonal anti-RhoA (clone 12, Abcam ab54835) and anti-Rac1 (clone 102, BD Biosciences 610651) antibodies that do not recognize the glycosylated isoforms of these proteins. *β*-Actin was used as a loading control using an anti-actin antibody produced in rabbits (A2066, Sigma) [28].

### Spore susceptibility to sodium dichloroisocyanurate

The susceptibility of spore suspensions to sodium dichloroisocyanurate (NaDCC) was determined using a dilution-neutralization assay [29,30]. To this end, 10^7^ spores were mixed with 0.3 g l^−1^ BSA and exposed to 1,000 p.p.m. NaDCC for 5 min at room temperature. Aliquots of these suspensions were transferred to tubes containing 0.5% sodium thiosulphate as a neutralizing agent and allowed to settle for 5 min at room temperature. Subsequently, three decimal dilutions were spread onto Brucella agar plates supplemented with 0.01% TA in duplicate. After 48 h of incubation, the number of c.f.u. per millilitre was recorded. This test was performed three times for each strain. Disinfectants with a logarithmic reduction factor (LRF) ≥5 are considered effective [31].

### Statistical analyses

Quantitative data were expressed as mean±sd. Statistical comparisons between means were conducted using one-way ANOVA followed by Tukey’s multiple comparison tests. Statistical significance was set at *P*<0.05.

## Results

### All ST exhibited comparable levels of spore adhesion, motility, biofilm formation ability and spore sensitivity to NaDCC

All the tested strains showed similar spore adhesion to human epithelial cells, ranging from 83% to 87 % (Fig. S1A, available in the online Supplementary Material). Likewise, all strains displayed equivalent swarming and swimming motility halos (Fig. S1B). Similar outcomes were obtained regarding biofilm formation at 24, 72 and 120 h (Fig. S1C). The LRFs of the strains ranged from 3.6 to 5.1, with LIBA-5757 (ST67) showing the highest resistance to NaDCC and LIBA-2811 (ST41) being the most sensitive. However, these values were statistically indistinguishable from those obtained for other STs (Fig. S1D).

### ST-specific SlpA profiles induced differential immune responses

The *slpA* gene exhibited substantial sequence diversity. According to PubMLST, ST01 strains LIBA-5700 and LIBA-5758 carried allele *slpA*116, while the remaining isolates harboured distinct alleles, including *slpA*10 (ST41), *slpA*30 (ST47) and *slpA*2 (ST67), as well as two novel full-length alleles (~*slpA*174 and ~*slpA*194) identified in ST154 and ST638, respectively (Table S1). The two ST01 isolates shared identical SlpA sequences (100% identity). Among the rest, the highest pairwise identity (58.2%) was observed between LIBA-5757 (ST67) and LIBA-5809 (ST638), while the lowest (36.3%) occurred between LIBA-7857 (ST47) and LIBA-6656 (ST154).

**Fig. 1.**
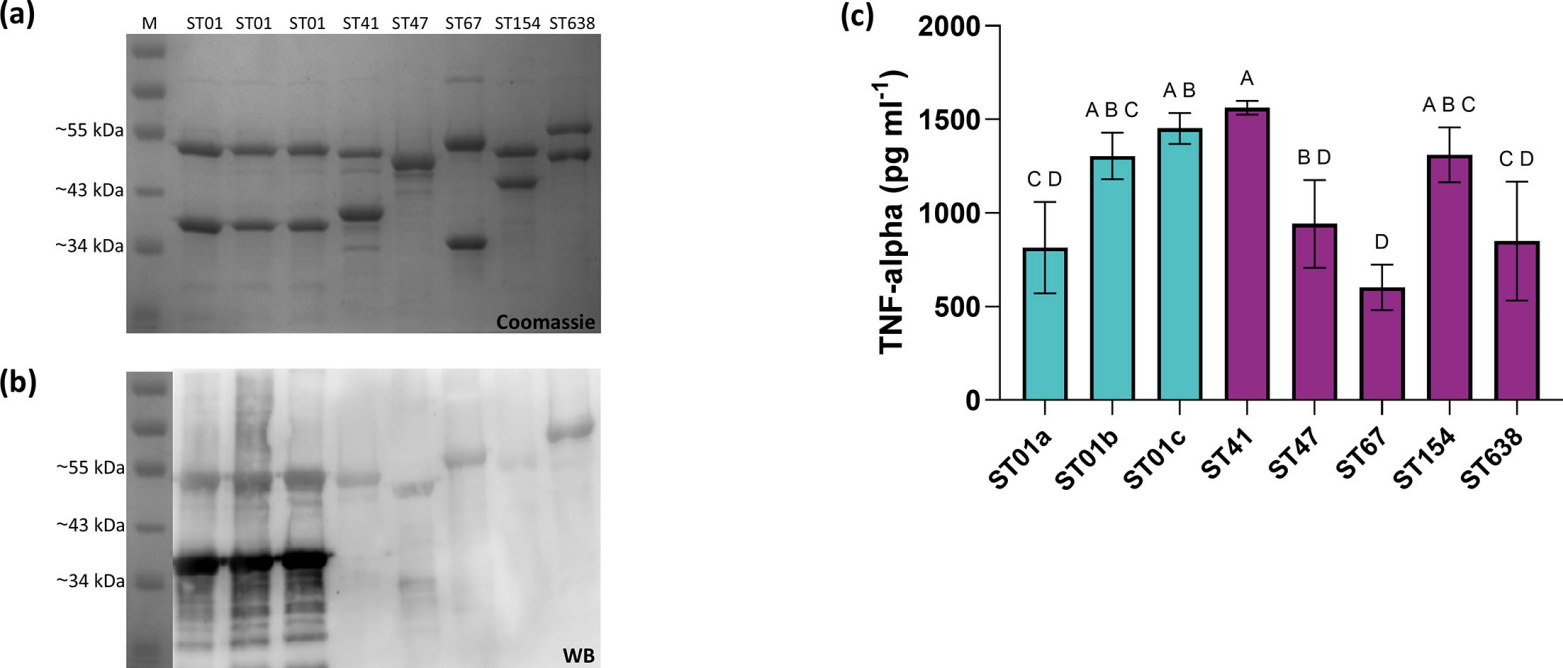
ST-specific SlpA profiles with distinct immunogenic properties and proinflammatory potential. (a) Coomassie-stained SDS-PAGE of SlpA preparations obtained for different *C. difficile* MLST Clade 2 strains by low-pH extraction. Lane M: PageRuler™ (Thermo Scientific) molecular weight marker; lanes designated as ST01 a/b/c correspond to strains R20291, LIBA-5700 and LIBA-5758, respectively. (b) WB of SlpA preparations probed with anti-LIBA5758-ST01 SlpA mouse antibodies. Immune complexes were revealed by chemiluminescence using Lumi Light Plus (Roche). (c) Twenty micrograms of SlpA preparations or glycine with Tris (in a ratio of 10:1) as a negative control C(-) were incubated with Raw 264.7 macrophages. The amount of TNF-*α* released to the supernatants after 12 h was quantified by ELISA using a mouse TNF-*α* kit (eBioscience). Bars designated as ST01 a/b/c represent strains R20291, LIBA-5700 and LIBA-5758, respectively. Error bars represent sd from three independent experiments (one-way ANOVA, with Tukey’s test, *P*<0.05).

SlpA preparations from these strains included high molecular weight (HMW, 45–52 kDa) and low molecular weight (LMW, 35–40 kDa) bands, except for LIBA-7857 (ST47), which showed a single HMW band (Fig. 1). Antibodies raised against SlpA from LIBA-5758 (ST01, 14) recognized both the HMW and LMW isoforms of the immunizing strain, while only recognizing the HMW isoforms of the other strains (Fig. 1).

The SlpA preparations of LIBA-2811 (ST41, 1,562 pg ml^−1^), followed by preparations of LIBA-5758 (ST01, 1,452 pg ml^−1^), LIBA-6656 (ST154, 1,311 pg ml^−1^) and LIBA-5700 (ST01, 1,305 pg ml^−1^) induced the higher secretion of macrophage-derived TNF-*α* (Fig. 1). These values were significantly different from the one obtained for LIBA-5757 (ST67, 603 pg ml^−1^), the lowest TNF-*α* producer in the assay. LIBA-7857 (ST47, 942 pg ml^−1^), LIBA 5809 (ST638, 850 pg ml^−1^) and R20291 (ST01, 815 pg ml^−1^) produced intermediate cytokine levels, indistinguishable from the amount recorded for LIBA-5757, our ST67 strain (Fig. 1). These results revealed distinct SlpA profiles and immunogenicity among the tested strains, highlighting variations in immune response induction. Interestingly, given that the immune response elicited by ST01 strains was not uniform, they could not be distinguished from strains of other STs.

### Distinct toxin profiles with varying levels of TcdB synthesis and CPE kinetics not correlating with TcdB amounts

Analysis of the *tcdA* and *tcdB* sequences using DiffBase revealed consistent toxin subtype combinations in the ST01 isolates, which both harboured TcdA2.1 and TcdB2.1 (Table S1). In contrast, the remaining STs exhibited greater diversity, with combinations of TcdA*2* paired with either TcdB2 or TcdB7 subtypes. These included TcdA2.6/TcdB7.2 (ST41), TcdA2.4/TcdB2.1 (ST47), TcdA2.2/TcdB7.7 (ST67) and TcdA2.18/TcdB2.9 (ST638). The ST154 isolate carried TcdA2.16, but its tcdB subtype could not be resolved due to the presence of two divergent alleles and fragmentation in the assembly from short-read data.

**Fig. 2.**
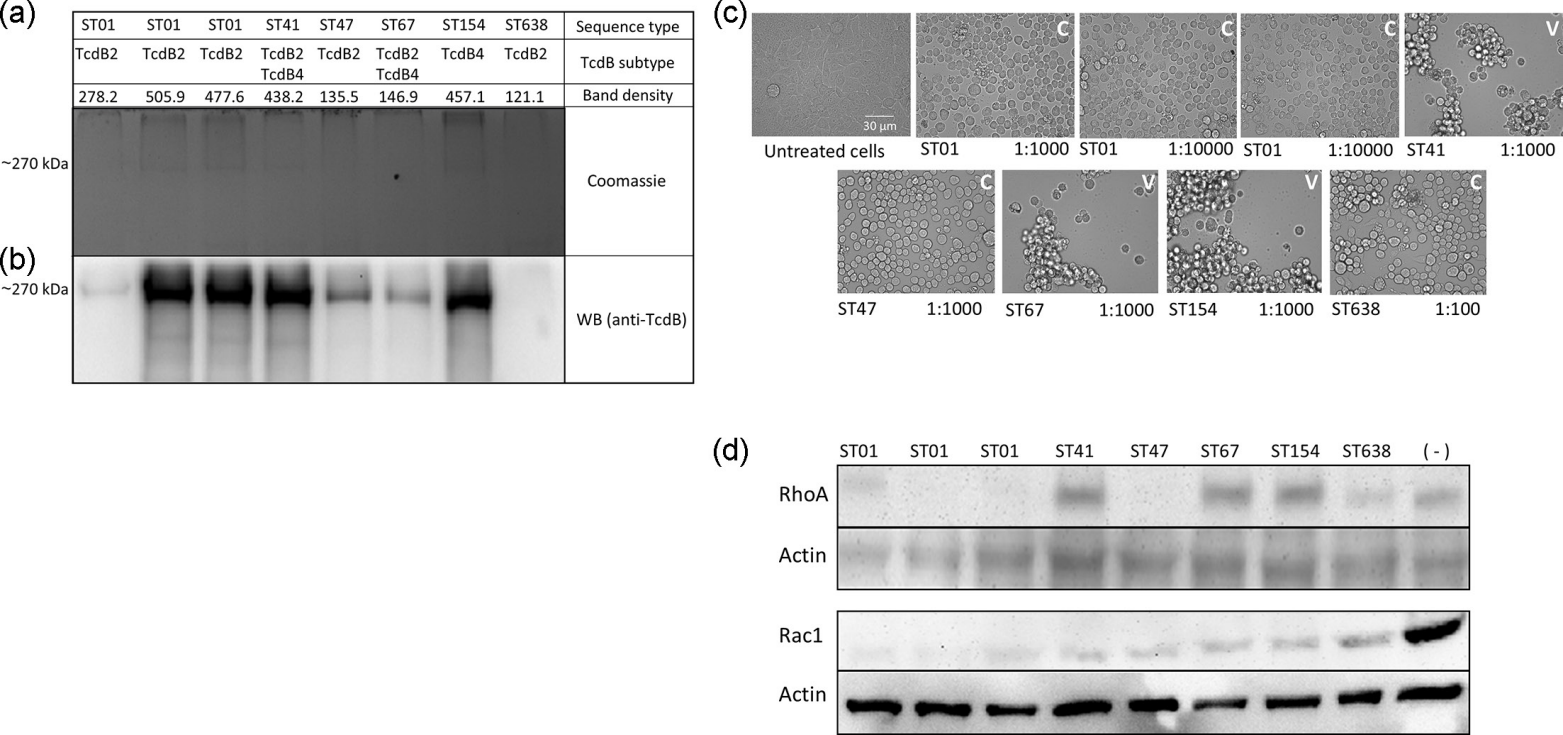
TcdB quantification and characterization of CPEs due to target glycosylation. (a) Proteins in cell-depleted supernatant were concentrated using StrataClean beads (Agilent Technologies), separated by 7.5% SDS-PAGE and visualized by Coomassie blue staining. The intensity of bands of ~270 kDa was measured using ImageJ. (b) To confirm the identity of the bands seen in the SDS-PAGE, a parallel SDS-PAGE gel was transferred to a PVDF membrane, probed with monoclonal antibody to TcdB (tgcBiomics), and the interaction complex was visualized by chemiluminescence using Lumi light Plus (Roche). (c) CPEs recorded 24 h after monolayers of HeLa cells were intoxicated with cell-free supernatants of the indicated strains. C, classic CPE; V, variant CPE. The decimal dilution in which CPE_50_ was observed is indicated next to each ST. (d) Monolayers of HeLa cells were intoxicated with cell-free supernatants for 24 h. Cells were lysed for subsequent evaluation of GTPase glycosylation by WB with antibodies that fail to recognize the glycosylated forms of RhoA and Rac1. As a loading control, membranes were revealed with a monoclonal antibody targeting actin. Untreated cells (-) were included as a control for unmodified proteins. Lanes or images designated as ST01 a/b/c correspond to strains R20291, LIBA 5700 and LIBA 5758, respectively.

Despite this genetic variability, our phenotypic analyses focused exclusively on TcdB, given its role as the principal *C. difficile* virulence factor and the variable presence of *tcdA* and *cdtAB* across the species. LIBA-5700 and LIBA-5758 (ST01), LIBA-2811 (ST41) and LIBA-6656 (ST154) exhibited significantly higher TcdB production (478–506 intensity units) compared with the other STs (121 to 278 intensity units), and LIBA-5809 (ST638) showed the lowest TcdB production (121 intensity units, Fig. 2). Our WB results primarily supported these densitometric measurements. However, it was noteworthy that LIBA-7857 (ST47) and LIBA-5757 (ST67) displayed strong WB signals despite their low band intensities in the SDS-PAGE gels (Fig. 2b).

TcdB secretion by the strains did not correlate with the magnitude of the recorded CPE, implying differences in the potencies of different toxin isoforms (Fig. 2). Specifically, LIBA-5700 and LIBA-5758 (ST01) exhibited significantly higher titres (1 : 10,000) compared with LIBA-2811 (ST41, 1 : 1,000) and LIBA-6656 (ST154, 1 : 1,000), which produced high toxin levels but showed similar titres to those of low TcdB producers (1 : 1,000) such as R20291, LIBA-7857 (ST47), LIBA-5757 (ST67) and LIBA-5809 (ST638, 1 : 100).

CPE kinetics also displayed variability among the strains. Cells exposed to supernatants from the highly toxin-producing ST01 strains LIBA-5700 and LIBA-5758 exhibited altered morphology as early as 3 h after exposure. In contrast, R20291 (ST01) and LIBA-7857 (ST47) required 4 h to induce morphological changes. Despite producing lower TcdB levels than LIBA-2811 (ST41) and LIBA-6656 (ST154), LIBA-5757 (ST67) caused cell alterations 2 or 3 h earlier, with CPE beginning at 7–8 h. In contrast, cells intoxicated with LIBA-5809 (ST638) took ~13 h to display CPE and retained neurite-like protrusions, even after 24 h of exposure. Altogether, these results indicate that ST01 and non-ST01 strains exhibit variability in TcdB production and CPE kinetics.

Distinct CPE patterns were observed among the strains, with ST01, ST47 and ST638 strains inducing a classical, arborizing CPE. In contrast, the tested ST41, ST67 and ST154 strains caused a variant, sordellii-like effect characterized by cell rounding, clumping and detachment. These differences in CPE patterns were consistent with the glycosylation patterns induced by the strains in HeLa cells, as the former subgroup of strains showed glycosylation of both RhoA and Rac1, and the latter only glycosylated Rac1 (Fig. 2c, d).

## Discussion

We comprehensively examined phenotypic and genomic differences across multiple STs from the *C. difficile* MLST Clade 2. All the STs tested displayed consistent spore adherence, similar swarming and swimming motility patterns, a shared tendency to form biofilms and comparable spore resistance to NaDCC. Therefore, these traits are unlikely to be the primary determinants of differences in pathogenicity. In contrast, a few phenotypic differences were observed, including strain-specific SlpA profiles uniquely recognized by mouse antibodies. However, the antibody reactivity observed in the two ST01 isolates did not differ from that of the strains of other STs. Another notable discrepancy among the strains was in TcdB production, where our analyses revealed that toxin identity, rather than quantity, influences toxin potency.

Clade 2 strains exclusively express TcdB2 or TcdB4, with ST67 and ST41 being capable of expressing either toxin subtype [32,33]. The primary receptor of TcdB2 and TcdB4 is the tissue factor pathway inhibitor (TFPI) [32]. Given that TFPI is widely distributed in glandular crypts, endothelial cells, enterocytes and other cell lines [32], the broader range of susceptible cells may partly explain the increased tissue damage distinguishing Clade 2 strains. Moreover, both *ex vivo* and animal experiments have demonstrated that strains expressing TcdB2 induce stronger proinflammatory activity [34], potentially aggravating pathology. This difference has been attributed to the faster internalization and auto-processing of TcdB2 within target cells, leading to a more efficient release of the enzymatically active domain [34,35].

Studies comparing the cytotoxic effects induced by TcdB2 from strains ST01 with those exerted by Clade 2 strains expressing TcdB4, such as ST67 strains, indicate that both subtypes can differentially glycosylate the GTPases Rac1 and Cdc42, as well as Rap1, Rap2 and R-Ras. However, of the two forms, only TcdB2 targets RhoA [34], whose glycosylation triggers the disruption of tight and adherent junctions and disestablishes the actin cytoskeleton to induce a classic, arborizing CPE [34,36, 37] Glycosylation of R-Ras by TcdB4, instead, leads to RhoA activation [36] and inactivation of integrins, resulting in the cell detachment that characterizes the so-called variant CPE [34,36, 38, 39].

It should be noted that some of the phenotypes analysed, such as motility, biofilm formation and toxin production, may show heterogeneity because of phase variation mechanisms [40,42], which may simultaneously influence multiple phenotypes. For instance, it has been shown that the transcription of genes involved in the late stages of flagellar production promotes the synthesis of *C. difficile* glycosylating toxins [41]. Phenotypic differences may be observed even across isolates from the same ST, as was recently reported for ST01 strains, which induce a spectrum of disease severity in a murine model. This characteristic was attributed to a 69 bp deletion in *cdtR*, a regulator of CDT and PaLoc expression, thus decreasing virulence without affecting colonizing capacity and persistence of the strains in the intestinal tract [18].

Overall, our data support therapeutic strategies targeting specific TcdB isoforms, possibly through vaccines or antibodies, and interventions aiming to modulate the immune response to SlpA.

## Supplementary material

10.1099/acmi.0.000994.v3 Uncited Supplementary Material 1.

## Abbreviations

BHI: brain heart infusion
BHI-CY: BHI broth supplemented with 0.1% cysteine and 0.5% yeast extract
CDI: *Clostridioides difficile* infection
CPE: cytopathic effect
CV: crystal violet
LRF: logarithmic reduction factor
NaDCC: sodium dichloroisocyanurate
ST: sequence type
TA: taurocholate
TFPI: tissue factor pathway inhibitor
TYT: Tryptose, Yeast extract and Thioglycollate
WB: Western blotting
