# Supplementary material for: Allele-linked divergence in SlpA and TcdB drives distinct immune and cytotoxic responses that distinguish ST01 from non-ST01 strains in Clade 2 Clostridioides difficile
Source: Access Microbiol. 2025 Sep 10;7(9):000994.v3. doi: 10.1099/acmi.0.000994.v3 (PMC12451308; doi:10.1099/acmi.0.000994.v3)
Supplement: Uncited Supplementary Material 1. [file acmi-7-00994-s001.pdf]

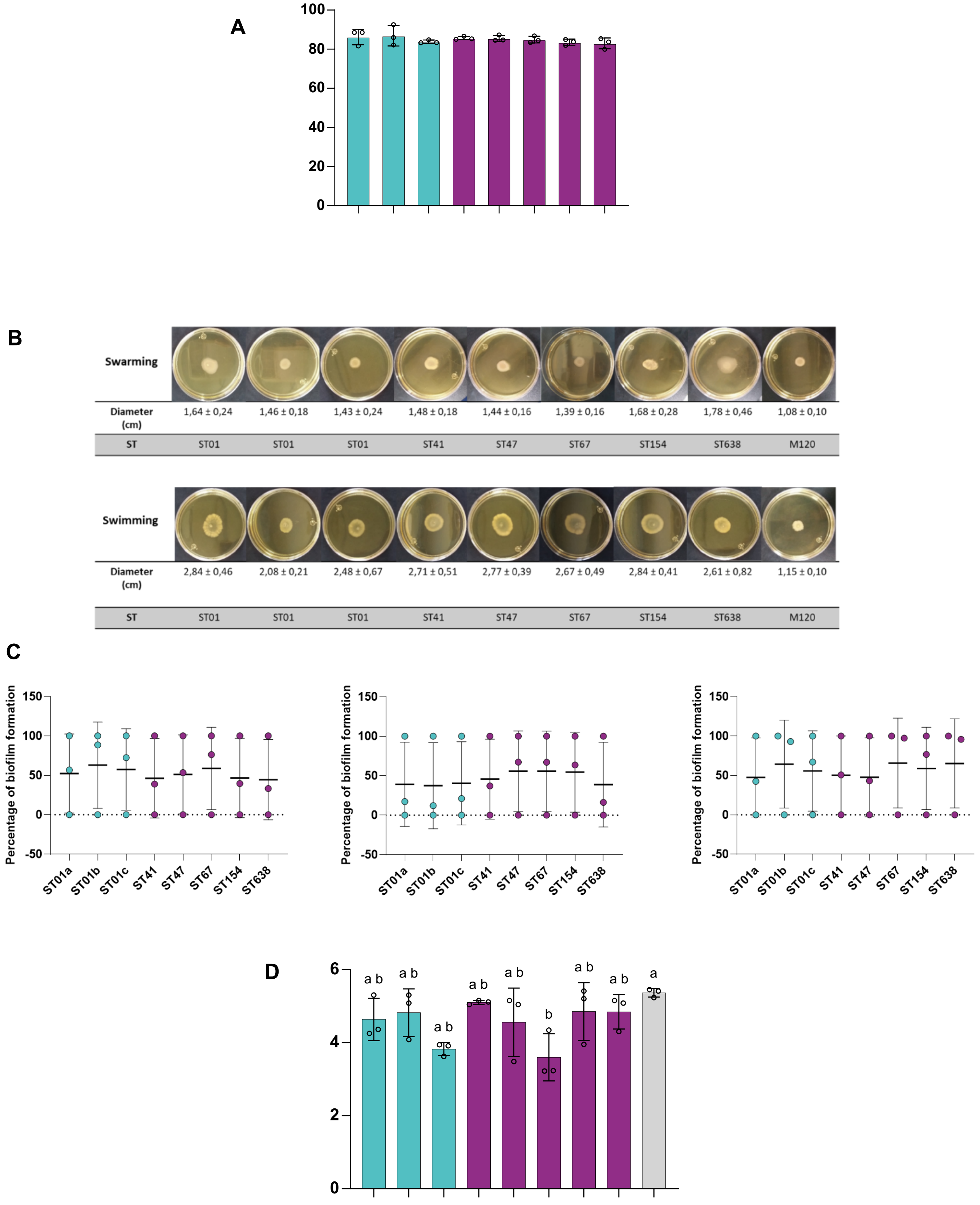
A
100
80
60
40
20
0
B
Swarming
Diameter (cm)
1,64 ± 0,24
1,46 ± 0,18
1,43 ± 0,24
1,48 ± 0,18
1,44 ± 0,16
1,39 ± 0,16
1,68 ± 0,28
1,78 ± 0,46
1,08 ± 0,10
ST
ST01
ST01
ST01
ST41
ST47
ST67
ST154
ST638
M120
Swimming
Diameter (cm)
2,84 ± 0,46
2,08 ± 0,21
2,48 ± 0,67
2,71 ± 0,51
2,77 ± 0,39
2,67 ± 0,49
2,84 ± 0,41
2,61 ± 0,82
1,15 ± 0,10
ST
ST01
ST01
ST01
ST41
ST47
ST67
ST154
ST638
M120
C
Percentage of biofilm formation
150
100
50
0
-50
ST01a
ST01b
ST01c
ST41
ST47
ST67
ST154
ST638
D
6
4
2
0
a b
a b
a b
a b
a b
b
a b
a b
a

Supplementary Figure 1. Strains from various MLST Clade 2 STs show similar adherence to human intestinal epithelial cells, motility traits, biofilm formation ability, and susceptibility to NaDCC. (A) Spore adherence to Caco-2 cells was determined quantitatively by plate counts. (B) Soft BHI agar plates were spot-inoculated and stab-inoculated with 4 µL of OD-adjusted bacterial cultures to assay swarming and swimming motility, respectively. Growth diameters in cm were recorded after 48 h. The non-motile strain M120 was included as a negative control (C-). (C) Diluted overnight cultures grown in BHIS supplemented with 0.1 M glucose were inoculated in triplicate in 24-well plates. Uninoculated culture medium was used as a (C-). The amount of biomass adhered to the plates after the indicated periods was estimated by quantitation of extracted crystal violet. (D) The inhibitory activity of NaDCC (1000 ppm) on spores ($1x10^7$ spores/mL) from eight different *C. difficile* MLST Clade 2 strains and the reference laboratory strain CD630 was tested in triplicate using a dilution-neutralization method. Results obtained were expressed as average log10 reduction factors (LRF), where an LRF = 5 indicates a 99.999% reduction in the original number of endospores. All results represent the average of three independent experiments, and error bars indicate standard deviations. No statistically significant differences were found at $p < 0.05$ (One-way ANOVA with Tukey's tests). ST01 a/b/c correspond to strains R20291, LIBA-5700, and LIBA-5758, respectively

Supplementary Table 1. Allelic profiles of *slpA*, *tcdA*, *tcdB*, and *cdtAB* in clinical Clade 2 ST01 and non-ST01 *Clostridioides difficile* strains

Strain	ST	*slpA*[a]	*tcdA*[b]	*tcdB*[b]	*cdtA*[a]	*cdtB*[a]
LIBA-5700<br>LIBA-5758	ST01	116	A2.1	B2.1	1	1
LIBA-2811	ST41	10	A2.6	B7.2	8	19
LIBA-7857	ST47	30	A2.4	B2.1	9	18
LIBA-5757	ST67	2	A2.2	B7.7	8	20
LIBA-6656	ST154	~174	A2.16	NA[c]	~9	~38
LIBA-5809	ST638	~194	A2.18	B2.9	29	18

[a]: ~: Novel full-length allele similar to allele n (PubMLST)
[b]: Best hit in Diffbase
[c]: NA: Not classifiable because the strain has two *tcdB* alleles
